# Massive Acetaminophen Overdose

**DOI:** 10.7759/cureus.9262

**Published:** 2020-07-18

**Authors:** Thor S Stead, Jae Yun Jeong, Latha Ganti, Jose Rubero

**Affiliations:** 1 Emergency Medicine, Alpert Medical School of Brown University, Providence, USA; 2 Biochemistry & Molecular Biology, Brown University, Providence, USA; 3 Emergency Medicine, Envision Physician Services, Nashville, USA; 4 Emergency Medicine, University of Central Florida College of Medicine/Hospital Corporation of America Graduate Medical Education Consortium of Greater Orlando, Orlando, USA; 5 Emergency Medical Services, Polk County Fire Rescue, Bartow, USA; 6 Emergency Medicine, University of Central Florida College of Medicine, Orlando, USA

**Keywords:** acetaminophen, toxicology, poisoning

## Abstract

The authors present a case of a fatal intentional acetaminophen (APAP) overdose and remind the physician how ubiquitous the drug is. This case presentation highlights the clinical presentation and treatment options for APAP overdose in unresponsive patients. In cases of massive APAP overdose (> 300 µg/ml plasma at four hours post-ingestion), prompt administration of N-acetylcysteine (NAC) and early hemodialysis are indicated.

## Introduction

Acetaminophen (APAP) is the most widely used over-the-counter pain reliever and antipyretic medication around the world [1,2]. However, high doses of APAP are hepatotoxic and are the most common cause of drug-induced liver failure in the United States (US) [3-5]. APAP overdoses in the United States account for 50,000 ED visits per year, over 10,000 hospitalizations, and approximately 100 deaths [2]. The majority of APAP overdose cases are a result of intentional self-harm; however, overdose can also be due to a lack of consumer knowledge. Unintentional overdoses often occur because the victim was unaware that APAP was an ingredient in a combination drug they were taking or because of the belief that over-the-counter drugs are universally benign [5]. If APAP overdose is caught early (< eight hours after ingestion), it is usually reversible via gastric decontamination and administration of the antidote N-acetylcysteine (NAC) [6,7].

## Case presentation

A 53-year-old male with unknown past medical history was brought in via ambulance after his neighbor called due to the patient being unresponsive. The patient was found by emergency medical services (EMS) with multiple bottles of empty acetaminophen around him on the bathroom floor and self-inflicted wounds on his wrist suggestive of a possible suicide attempt. He was in cardiac arrest with pulseless electrical activity (PEA). He received naloxone en route by the paramedics. Return of spontaneous circulation (ROSC) was achieved within 10 minutes of cardiopulmonary resuscitation. He had agonal breathing and pupils that were 3 mm, round, and sluggish bilaterally.

Upon arrival at the ED, the patient was started on vasopressors and intubated. In the ED, the temperature was 89.9^0^F, with a mean arterial pressure of 50 mmHg on norepinephrine infusion, a pulse between 90 and 100 beats per minute, a respiratory rate of 20 (ventilator rate), and oxygen saturation of 94% on 100% FiO2. Laboratory analysis was remarkable for a white blood cell count of 33 x 103/mm^3^ with neutrophilic predominance. Hepatic panel showed transaminitis with aspartate aminotransferase 1256 units/L (normal range 10-37) and alanine aminotransferase 232 units/L (normal range 12-78). Lactic acid level was 12 mg/dL. The patient had an anion gap metabolic acidosis with bicarbonate of 11 mmol/L, and evidence of acute kidney injury (AKI), with serum creatinine 7 mg/dL and creatine protein kinase >1000 units/L. Arterial blood gas revealed a pH of 6.88, CO_2 _of 51 mmHg, pO_2_ of 259 mmHg. The patient had an elevated troponin of 35 ng/mL, but cardiac echocardiogram revealed a normal left ventricular ejection fraction of 55% (Tables 1-4).

**Table 1:** Complete blood count profile WBC, white blood cell count; RBC, red blood cell count; Hgb, hemoglobin; Hct, hematocrit; MCV, mean corpuscular volume; MCH, mean corpuscular hemoglobin; MCHC, mean corpuscular hemoglobin concentration; RDW, red cell distribution width; MPV, mean platelet volume

WBC (4.0 - 12.0 K/mm^3^)	32.82 *H
RBC (4.6 - 6.0 M/mm^3^)	4.66
Hgb (14.0 - 18.0 gm/dL)	14.3
Hct (42.0 - 52.0 %)	43.4
MCV (80.0 - 100.0 fL)	93.1
MCH (27.0 - 31.0 pg)	30.7
MCHC (32 - 37.5 g/dL)	32.9
RDW (12.0 - 14.0 %)	14.1 H
Plt Count (130 - 400 K/mm^3^)	282
MPV (7.0 - 10.0 fL)	10.3
Segmented Neutrophils (38.0 - 74.0 %)	69
Band Neutrophils (5.0 - 11.0 %)	14 H
Lymphocytes (20 - 45 %)	6L
Monocytes (3 - 10 %)	10
Atypical Lymphocytes (0 - 1 %)	1

**Table 2:** Chemistry profile BUN, blood urea nitrogen; GFR, glomerular filtration rate; AST, aspartate aminotransferase; ALT, alanine aminotransferase

Sodium (136 - 145 mmol/L)	148 H
Potassium (3.7 - 5.1 mmol/L)	3.5 L
Chloride (98 - 107 mmol/L)	102
Carbon Dioxide (21 - 32 mmol/L)	11 L
Anion Gap	38.5
BUN (7 - 18 mg/dL)	30 H
Creatinine (0.55 - 1.3 mg/dL)	4.30 H
Estimated GFR mL/min	15
BUN/Creatinine Ratio	7
Glucose (74 - 106 mg/dL)	176 H
Calcium (8.4 - 10.1 mg/dL)	7.2 L
Magnesium (1.8 - 2.5 mg/dL)	7.2 H
Total Bilirubin (0.2 - 1.5 mg/dL)	2.7 H
AST (10 - 37 unit/L)	1256 H
ALT (12 - 78 unit/L)	232 H
Total Alk Phosphatase (45 - 117 unit/L)	89
Total Protein (6.4 - 8.2 g/dL)	6.2 L
Albumin (3.4 - 5.0 g/dL)	3.6

**Table 3:** Arterial blood gas POC ABG, point-of-care arterial blood gas test

Puncture site	R Radial
POC ABG pH (7.35 - 7.45)	6.88L
POC ABG pCO2 (35 - 45 mmHg)	51.8 H
POC ABG pO2 (80 - 105 mmHg)	259 H
ABG PO2/FiO2 Ratio	259
POC ABG HCO3 (22 - 26 mmol/L)	9.7 L
ABG Total CO2 (23 - 27 mmol/L)	11 L
ABG O2 Saturation (95 - 98 %)	99 H
POC ABG Base Excess (-2 - 3 mmol/L)	-23 L
Allen Test	Pass
Set Respiration Rate (/min)	20
O2 Delivery Device	Vent
Vent Mode	CMV
POC FiO2 (%)	100
Tidal Volume (mL)	500
POC PEEP (cmH2O)	5

**Table 4:** Toxicology screen

Opiates	Negative
Methadone	Negative
Barbituates	Negative
Phencyclidine	Negative
Amphetamines	Negative
Benzodazepines	Negative
Cocaine	Negative
Cannabinoids	Negative
Acetaminophen (10 - 30 mcg/ml)	> 300 *H
Alcohol, Quantitative (<5.0 mg/dl)	< 3 L
Salicylates (4 - 20 mg/dL)	4.8

**Figure 1:**
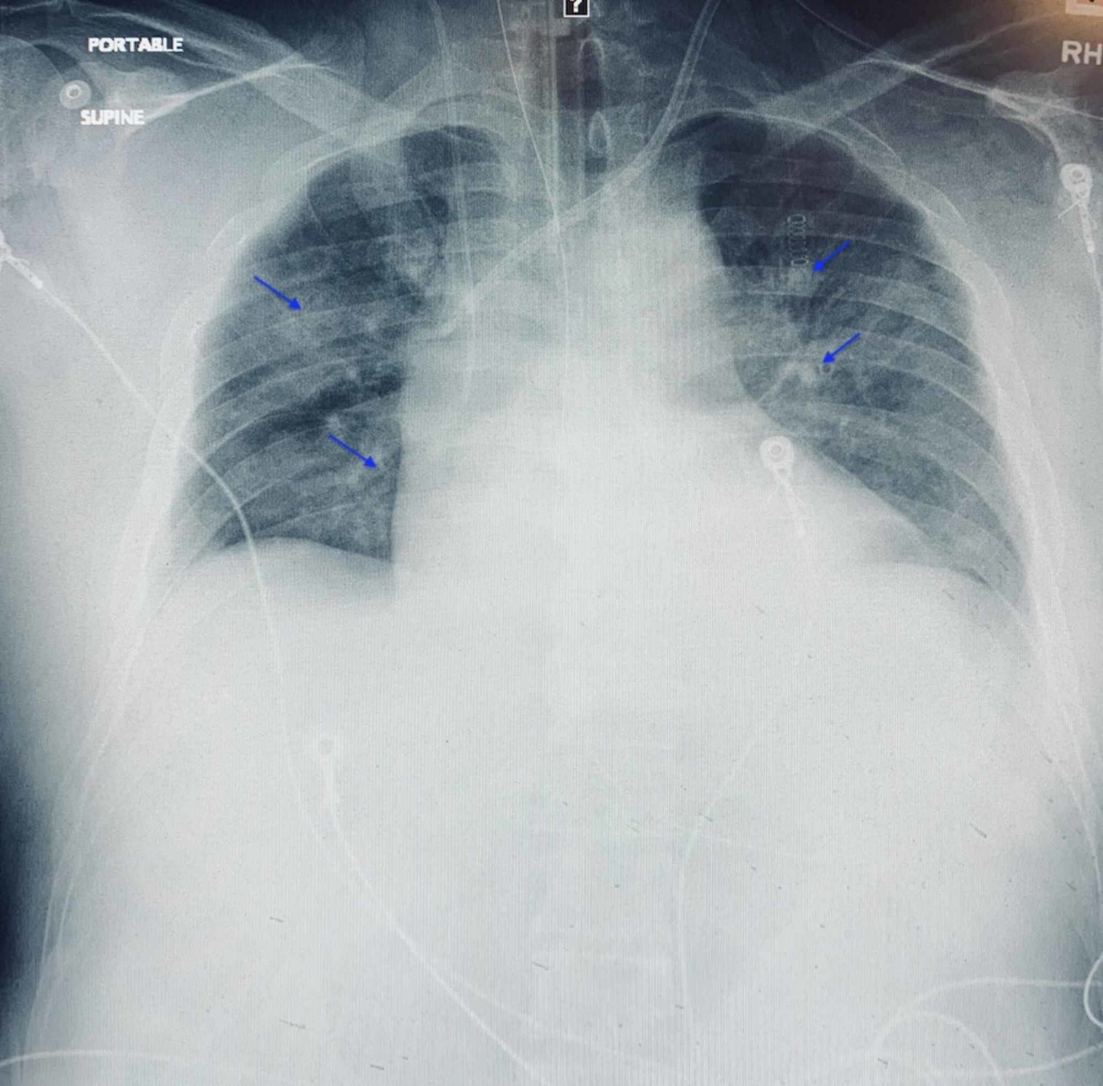
Chest radiograph demonstrating mild bilateral perihilar airspace opacities (arrows)

The acetaminophen level was >300 mcg/mL (normal range 10-30). Toxicology screen was negative for cocaine, barbiturates, methadone, phenylcyclidine, amphetamines benzodiazepines, or cannabinoids. Foley insertion revealed anuria since admission. Chest radiograph revealed mild bilateral perihilar airspace opacities (Figure 1).

A central line was placed in the right internal jugular vein, a trialysis catheter placed in the left internal jugular vein, and a right femoral arterial line was placed. The poison control center was called and NAC was initiated. Four ampules of bicarbonate were given and a bicarbonate infusion was started. Additional pressors including vasopressin, epinephrine, and neosynephrine were required. Active body warming was initiated and nephrology was consulted for continuous renal replacement therapy (CRRT). Aggressive intravenous fluids were initiated (>4L) without significant improvement. Approximately 12 hours later, the patient went into cardiopulmonary arrest and expired.

## Discussion

APAP is primarily metabolized by adding a glucuronide or sulfate to the hydroxyl group, forming nontoxic mercaptate conjugates that are excreted. Above the toxic threshold, APAP administration produces NAPQI (N-acetyl-p-benzoquinoneimine), a toxic compound which binds proteins, resulting in hepatocyte necrosis. This occurs when glutathione concentration within the liver decreases to <30% of normal levels [5].

**Figure 2:**
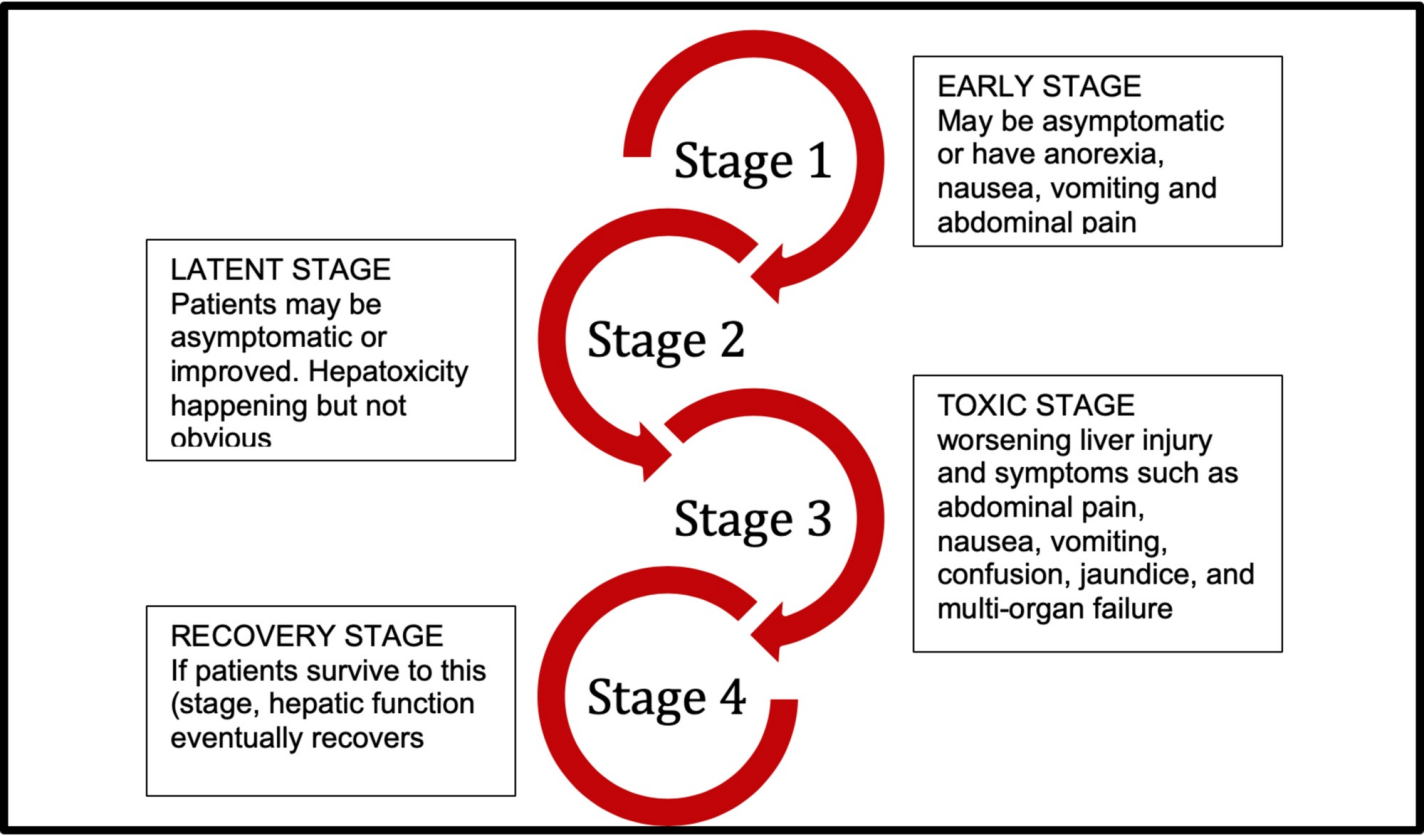
Stages of acetaminophen toxicity

The range of symptoms in APPA overdose can be grouped into four stages (Figure 2). 

**Figure 3:**
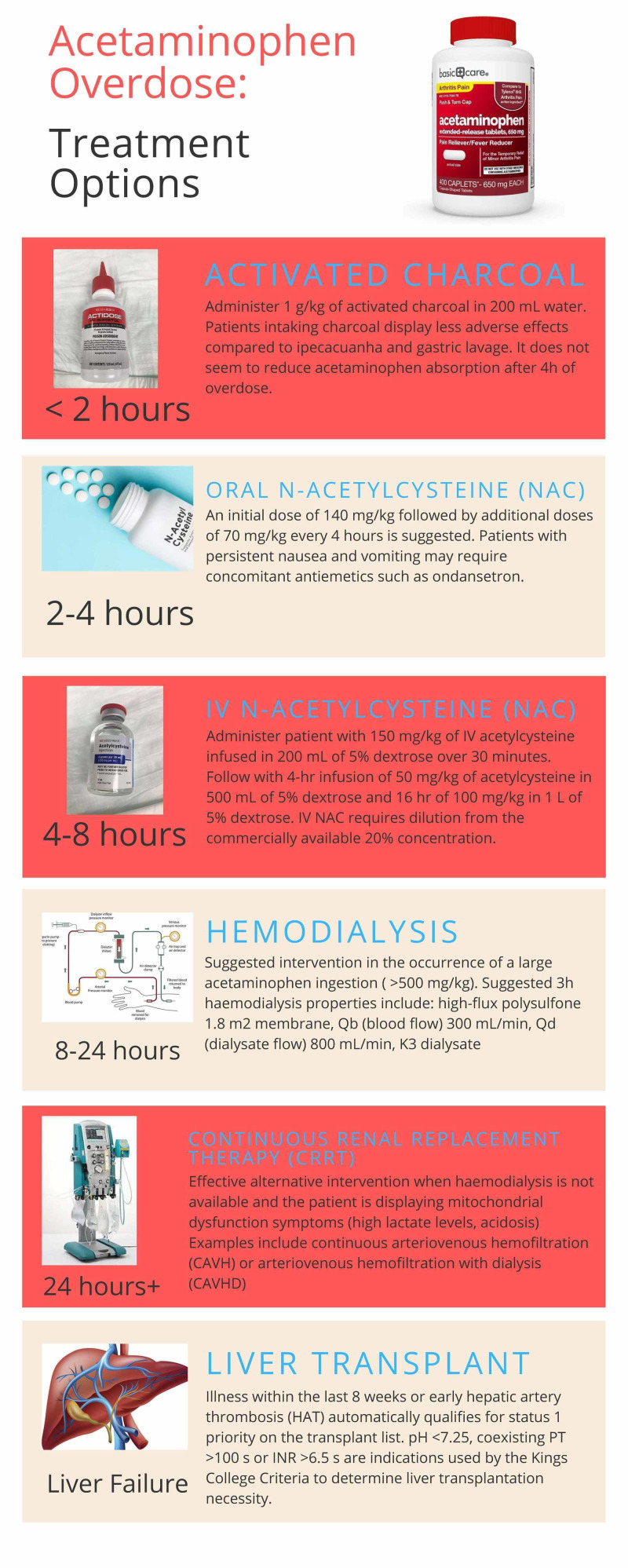
Acetaminophen poisoning treatment options

Interventions for APAP overdose include gastrointestinal decontamination through oral activated charcoal, NAC administration, and dialysis (Figure 3).

Activated charcoal should only be given if the ingestion occurred within the last two hours. It is administered as 1 g/kg of active charcoal in 200 mL water. NAC is available in both oral and intravenous formulations. In the ED, the IV formulations are most commonly used. IV NAC is given as 150 mg/kg infused in 200 mL of 5% dextrose over 30 min, followed by a four‐hour infusion of 50 mg/kg of acetylcysteine in 500 mL of 5% dextrose and a 16-hour of 100 mg/kg in 1 L of 5% dextrose. If serum APAP concentration is <10 micrograms/mL and transaminase concentrations are normal, then acetylcysteine therapy can be discontinued and discharge can be considered [5].

**Figure 4:**
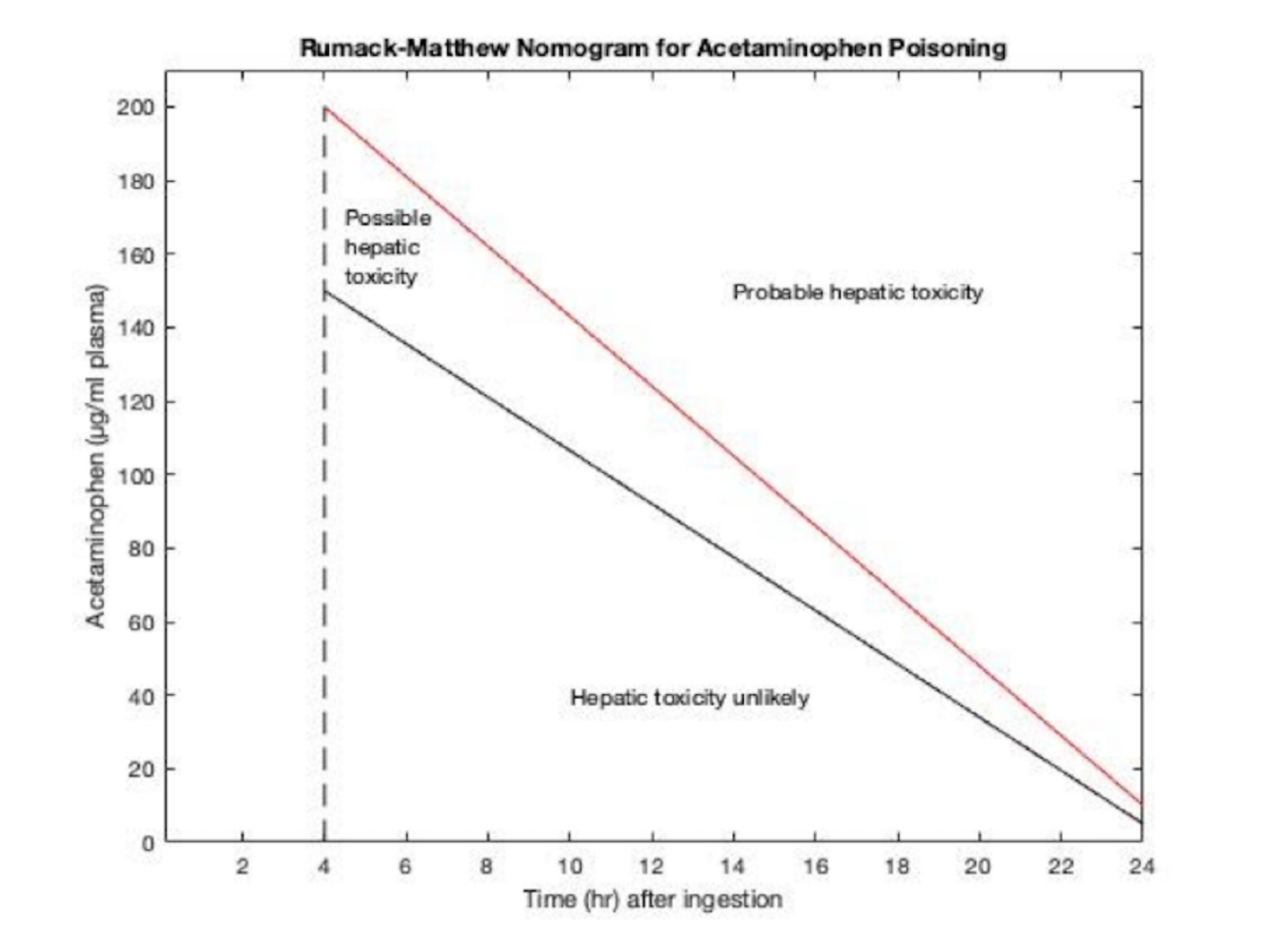
Acetaminophen nomogram adapted from Rumack BH, Matthew H: Pediatrics 55:871, 1975

Treatment threshold for NAC antidote treatment is based on the Rumack-Matthew nomogram which plots APAP levels against time (Figure 4).

Recent studies show that IV NAC treatment administered at lower doses reduces potential side effects such as nausea and allergy [8]. Hemodialysis is recommended for patients with extreme plasma acetaminophen concentrations (> 200 µg/mL plasma at four hours post-ingestion) or displaying symptoms of mitochondrial dysfunction such as elevated lactate levels and acidosis [9,10]. CRRT is also suggested in severe cases in which patients display neurological, respiratory, or circulatory malfunctioning (encephalopathy or coma) as our patient did [11].

Patients lacking glutathione reserves such as those with chronic alcohol use are especially at risk, but prompt acetylcysteine administration can effectively prevent acute liver failure. Our patient had likely overdosed the night prior given he arrived in multiorgan failure with irreversible damage to the liver. The patient had an acetaminophen level more than 10x normal. Other complications such as cardiac arrest, respiratory failure, rhabdomyolysis, and septic shock developed, rendering interventions ineffective. 

Growth factors such as hepatocyte growth factor (HGF) are known to be crucial in liver regeneration, a continuous interest amongst researchers in their therapeutic application. However, higher concentrations of HGF in plasma were found in nonsurvivors as opposed to survivors, suggesting that interventions via other factors such as c-Met or TGF-β may be more effective for treatment [12]. In survivors, liver function is normally regained within three months.

## Conclusions

This case demonstrates the evaluation and management of late-stage APAP overdose in an unresponsive patient. Key takeaways include the rapid administration of CRRT in the presence of renal failure, administration of pressors for hypotension, IV NAC dosed according to the APAP nomogram, and intubation if unresponsive.
